# Effects of 4‐month treatment with glycocalyx dietary supplement on endothelial glycocalyx and vascular function after COVID‐19 infection

**DOI:** 10.1111/eci.70058

**Published:** 2025-04-24

**Authors:** George Pavlidis, Aikaterini Kountouri, Konstantinos Katogiannis, John Thymis, Panagiota Efstathia Nikolaou, Christina Chania, John Karalis, Gabriella Kostelli, Eleni Michalopoulou, Eleni Katsanaki, John Parissis, Hans Vink, Robert Long, Sotirios Tsiodras, Vaia Lambadiari, Ignatios Ikonomidis

**Affiliations:** ^1^ 2nd Department of Cardiology Attikon University Hospital, School of Medicine, National and Kapodistrian University of Athens Athens Greece; ^2^ 2nd Propaedeutic Department of Internal Medicine, Research Unit and Diabetes Center Attikon University Hospital, School of Medicine, National and Kapodistrian University of Athens Athens Greece; ^3^ Laboratory of Pharmacology School of Pharmacy, National and Kapodistrian University of Athens Athens Greece; ^4^ University Department of Emergency Medicine Attikon University Hospital, School of Medicine, National and Kapodistrian University of Athens Athens Greece; ^5^ GlycoCalyx Research Institute Alpine Utah USA; ^6^ 4th Department of Internal Medicine Attikon University Hospital, School of Medicine, National and Kapodistrian University of Athens Athens Greece

**Keywords:** arterial stiffness, coronavirus disease 2019, endothelial glycocalyx, fucoidan, glycocalyx dietary supplement, pulse wave velocity

## Abstract

**Background:**

Coronavirus disease 2019 (COVID‐19) has been associated with impaired endothelial and vascular function. We investigated whether intervention with glycocalyx dietary supplement (GDS), containing glucosamine sulfate and fucoidan, improves endothelial glycocalyx and vascular function after COVID‐19 infection.

**Methods:**

Fifty‐seven convalescent patients 14 days after mild‐to‐moderate COVID‐19 infection managed in an outpatient setting were randomized to receive GDS (*n* = 29) or placebo (*n* = 28) for 4 consecutive months. We measured at baseline and at 4 months: (a) perfused boundary region (PBR) of the sublingual microvessels with a diameter range of 4–25 μm, as a marker of endothelial glycocalyx integrity, (b) pulse wave velocity and augmentation index, (c) coronary flow reserve using Doppler echocardiography, and (d) malondialdehyde and protein carbonyls as oxidative stress markers.

**Results:**

Four months after treatment, patients who received GDS showed a greater reduction in PBR 4–25 μm (−6.8% vs. −1.3%), pulse wave velocity (−13.2% vs. −3%), augmentation index (−28.5% vs. −2.5%), malondialdehyde (−26% vs. −2.9%), protein carbonyls (−31.3% vs. −1%) and a greater increase in coronary flow reserve (12.9% vs. 1.6%) compared to placebo (*p* < .05). In the GDS group, the reduction in PBR 4–25 μm was associated with the corresponding decrease in pulse wave velocity (*r* = .31, *p* = .047), malondialdehyde, and protein carbonyls, as well as with the increase in coronary flow reserve (*r* = −.59, *p* = .008) at follow‐up. Post‐treatment, none of the patients under GDS reported post‐COVID symptoms compared to 21.4% of the patients under placebo.

**Conclusion:**

Four‐month treatment with GDS may improve endothelial glycocalyx and vascular function after COVID‐19 infection.

**Clinical Trial Registration:**

URL: https://www.clinicaltrials.gov. Unique identifier: NCT05185934.

## Graphical abstract

The present study aimed to investigate the effect of glycocalyx dietary supplement (GDS), containing glucosamine sulfate and fucoidan, on endothelial glycocalyx integrity and vascular function in subjects after mild‐to‐moderate COVID‐19 infection managed in an outpatient setting. Four‐month administration of GDS resulted in significant improvement in endothelial glycocalyx integrity, vascular function, and coronary microcirculation in conjunction with a substantial reduction in oxidative stress. These findings suggest that GDS administration for a short‐term period might mitigate or prevent potential subclinical COVID‐19‐related cardiovascular complications.

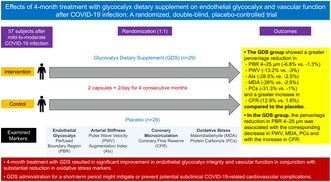

## 1 INTRODUCTION

Coronavirus disease 2019 (COVID‐19), an infectious disease caused by the novel severe acute respiratory syndrome coronavirus 2 (SARS‐CoV‐2), has been associated with many cardiovascular complications, such as arrhythmias, myocardial injury and myocarditis, heart failure, and thromboembolic events.^1^ Direct viral infection of endothelial and myocardial cells, proinflammatory cytokine storm during the clinical course of infection, and microangiopathy have been suggested as possible pathophysiological mechanisms of COVID‐19‐associated cardiovascular disease.^2^ In a previous study, we showed that SARS‐CoV‐2 infection causes endothelial and vascular dysfunction, increased oxidative stress and reduced cardiac performance that remain 4 months post‐infection, independently of disease severity.^3^ Interestingly, vascular dysfunction and impaired myocardial performance are partially restored at 12 months after initial COVID‐19 infection.^4^

Endothelial glycocalyx is a complex gel‐like mesh composed of glycosaminoglycans, glycolipids, glycoproteins, and proteoglycans that cover the luminal side of endothelial cells.^5^ This structure acts as a key regulator of vascular barrier permeability by preventing the direct contact of circulating blood cells with the endothelium.^6^ Inflammatory conditions, including COVID‐19 infection, are associated with impaired endothelial glycocalyx integrity leading to endothelial dysfunction.^3^ The degradation of glycocalyx is considered to be one of the earliest stages in the atherogenic processes and is an independent predictive marker of adverse outcomes in subjects without established cardiovascular disease.^7^ Moreover, the MYSTIC study revealed that glycocalyx integrity was a significant predictive marker in patients with COVID‐19.^8^

Glycocalyx dietary supplement (GDS) constitutes a novel promising dietary intervention that has been shown to preserve endothelial glycocalyx by supplying glucosamine sulfate, fucoidan, antioxidant enzymes, and additional major substrates for the synthesis of glycocalyx layer.^9^, ^10^ Emerging evidence from experimental and clinical studies suggests that GDS administration has a beneficial effect on glycocalyx integrity in several conditions, including chronic kidney disease,^9^ type 2 diabetes mellitus,^11^ as well as in older adults.^12^ Furthermore, in a recently published randomized controlled trial, we demonstrated that the addition of GDS to standard treatment with biological agents improved endothelial glycocalyx thickness and arterial stiffness in patients with psoriasis.^13^ However, the impact of GDS on endothelial glycocalyx, vascular function, coronary microcirculation, and oxidative stress in subjects after mild‐to‐moderate COVID‐19 infection has not yet been investigated.

In the present study, we hypothesized that GDS administration causes greater improvement in endothelial glycocalyx integrity, arterial stiffness, and coronary microcirculation, and decreases oxidative stress compared to placebo in individuals after COVID‐19 infection. Therefore, the main objective of our study was to investigate the changes in endothelial glycocalyx, arterial stiffness, as evaluated by pulse wave velocity (PWV) and augmentation index (AIx), coronary flow reserve (CFR) and oxidative stress markers, namely malondialdehyde (MDA) and protein carbonyls (PCs) after 4‐month treatment with GDS.

## 2 METHODS

### 2.1 Study population

**FIGURE 1:**
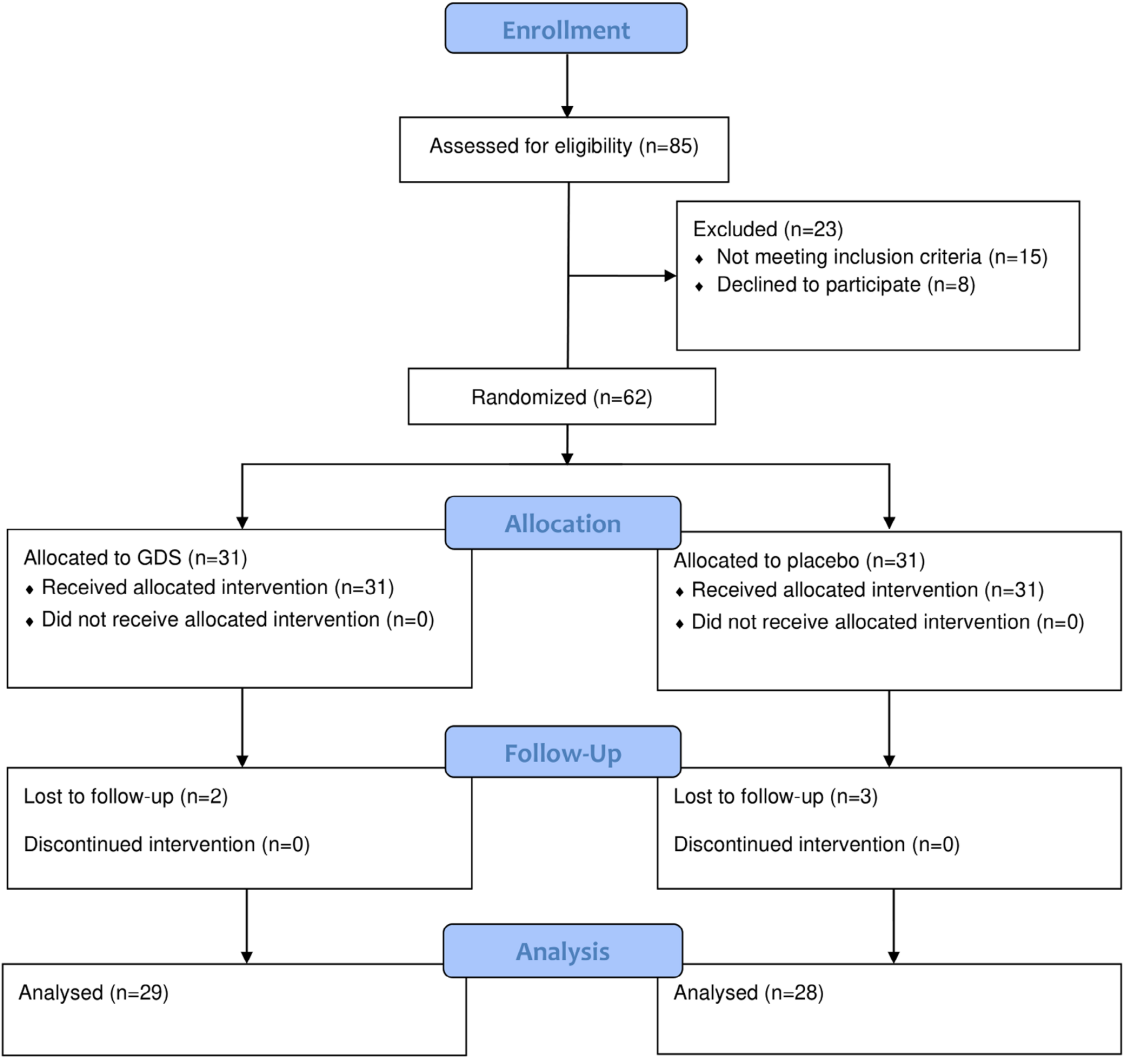
CONSORT 2010 flow diagram of the study progress. GDS, glycocalyx dietary supplement.

Eighty‐five patients who were referred to the outpatient COVID‐19 clinic of Attikon University Hospital, Athens, Greece, were assessed for eligibility by the attending physician (A.K.). Eligible participants were adults aged between 18 and 75 years old with mild‐to‐moderate COVID‐19 infection confirmed by real‐time reverse transcription polymerase chain reaction assay, who did not fulfil criteria for hospitalization according to the current guidelines of the National Institutes of Health.^14^ We chose to include patients with mild‐to‐moderate COVID‐19 infection and not hospitalized patients with moderate or severe infection to avoid the effects of excess inflammation and hypoxia due to persisting lung disease as early as 14 days after onset of COVID‐19 infection (baseline visit). Exclusion criteria included pregnancy and breastfeeding, a history of coronary artery disease, heart failure, moderate or severe valvular disease, primary cardiomyopathies, chronic obstructive pulmonary disease, asthma, chronic kidney disease, defined as an estimated glomerular filtration rate ≤60 mL/min/1.73 m^2^, severe liver disease, chronic inflammatory diseases, and active malignancies. The recruitment took place between June 2022 and April 2024. Out of 85 patients, 23 subjects were excluded owing to a history of coronary artery disease (*n* = 7), heart failure (*n* = 4), chronic kidney disease (*n* = 3), chronic inflammatory disease (*n* = 1) and unwillingness to participate in the study (*n* = 8). Therefore, 62 patients were randomized to receive GDS (*n* = 31) or placebo (*n* = 31). Of the 62 participants, 5 subjects did not complete the study protocol. More specifically, 2 subjects in the GDS group and 3 subjects in the placebo group were lost to follow‐up. Thus, 57 patients (29 subjects in the GDS group and 28 subjects in the placebo group) were finally included in the analysis. A comprehensive flowchart diagram of the study progress is depicted in Figure 1.

The investigation followed the guidelines and regulations of the Helsinki Declaration. The Institutional Review Board of Attikon University Hospital approved the study protocol (Approval number: 668/30‐11‐2021). Additionally, the study has been registered to ClinicalTrials.gov under ID number: NCT05185934. All subjects signed an informed consent form prior to their participation in the study.

### 2.2 Study design and protocol

In the current randomized, double‐blind, placebo‐controlled study, all participants were randomly assigned in a 1:1 ratio to receive 2 capsules at 8:00 am and 2 capsules at 6:00 pm per day, either of the GDS or the placebo, for 4 consecutive months. The assignment to a treatment regimen was conducted by an attending physician (G.P.) utilizing a table of random numbers derived from the online randomization software available at: http://www.graphpad.com/quickcalcs/index.cfm (accessed on June 1, 2022). Each participant underwent two outpatient visits, one at 14 days from COVID‐19 onset (baseline visit) and the other 4 months later (follow‐up visit). We chose the 4‐month intervention time frame as in our previous study we observed significant alterations in endothelial and cardiovascular function 4 months after COVID‐19 infection and to avoid the effects of the acute inflammatory effects of COVID‐19 infection on the cardiovascular bed.^3^ In both visits, all participants underwent assessment of endothelial glycocalyx and arterial stiffness, echocardiography examination to measure CFR, while blood samples were collected for evaluation of oxidative stress markers, namely MDA and PCs. The aforementioned examinations were performed by physicians who were blinded regarding the clinical data of participants. In addition, the presence of COVID‐19 symptoms related to the cardiovascular system, namely fatigue, dyspnoea, cough, and chest pain, were recorded at both baseline and follow‐up visit.

### 2.3 Composition of glycocalyx dietary supplement

The trade name of GDS is Endocalyx‐Pro™. This dietary supplement is produced and provided by Microvascular Health Solutions (Alpine, Utah, USA). It is a blend of 2475 mg per serving of 4 capsules. Each capsule contains glucosamine sulfate (375 mg), fucoidan (106.25 mg) extracted from the algae species Laminaria japonica, hyaluronic acid (17.5 mg), a blend of superoxide dismutase and polyphenols (120 mg) extracted from olive, artichoke leaves and red and white grapes, microcrystalline cellulose (130 mg) and silicon dioxide (2 mg). The placebo capsule mimics the Endocalyx‐Pro™ capsules and consists of Nu‐MAG (10 mg), which is a blend of 4 ingredients (rise flour extract, rice hulls, gum Arabic, and sunflower oil), white rice flour (830 mg), magnesium stearate (10 mg) and microcrystalline cellulose (130 mg).

The capsules are administered orally and can be received with water, regardless of food intake. Throughout the follow‐up period, no adverse effects were reported with regard to the use of GDS or placebo.

### 2.4 Primary and secondary outcomes

The primary outcome of the current study was to investigate the changes in endothelial glycocalyx integrity after four‐month administration of GDS compared to placebo. The secondary outcomes were to assess the changes in arterial stiffness, coronary microcirculation, and oxidative stress at 4‐month follow‐up.

### 2.5 Endothelial glycocalyx measurement

The perfused boundary region (PBR, μm) of the sublingual microvasculature with a lumen diameter ranged from 4 to 25 μm was assessed using Sidestream Darkfield camera (Microscan, GlycoCheck, Microvascular Health Solutions Inc., Salt Lake City, Utah, USA).^15^ This non‐invasive technique allows us to estimate the endothelial glycocalyx integrity. The Sidestream Darkfield camera is placed under the tongue and records over 3000 vascular segments in approximately 3 min. After image acquisition, the width of the median red blood cell column and the total perfused diameter of the microvessel segments are evaluated utilizing GlycoCheck software. PBR value is calculated using the following equation: (red blood cell perfused lumen diameter − width of the median red blood cell column)/2.^16^ Subsequently, software calculates the PBR of vessels between 4 and 9 μm, 10 and 19 μm, and 20 and 25 μm and the mean 4–25 μm. The normal value of PBR is reported 2 ± .2 μm.^6^ An increased PBR value indicates a greater penetration of red blood cells towards the endothelial surface and reflects a damaged endothelial glycocalyx. On the contrary, low PBR value reflects a preserved endothelial glycocalyx integrity. Inter‐ and intra‐observer variabilities of PBR assessment were 5.2% and 4.3%, respectively.^7^ Microvascular vessel density was calculated from the number of microvessel measurement sites containing erythrocytes and representing 10 μm of length. The cumulative microvessel segment length was divided by the total recorded area to determine total microvascular density.^16^

### 2.6 Arterial stiffness assessment

Carotid to femoral PWV (m/s) was evaluated using tonometry (Complior SP, Alam Medical, Vincennes, France) according to a previously published methodology.^17^ Normal value of PWV is reported to be <10 m/s for the indirect method (common carotid artery − common femoral artery × .8).^18^ AIx (%) was calculated by oscillometry using the following formula: [(late systolic peak pressure − early systolic peak pressure)/pulse pressure] × 100.^13^

### 2.7 Coronary flow reserve measurement

Coronary flow velocities in the left anterior descending coronary artery were obtained using colour‐guided pulsed‐wave Doppler from a long‐axis apical view with a 7 MHz transducer. Peak diastolic velocity of the coronary flow wave at rest and during hyperaemia was assessed after adenosine infusion (140 μg/kg/min) for 3 min. CFR was evaluated as the ratio of hyperaemic to baseline resting flow velocity.^3^ Inter‐ and intra‐observer variabilities of CFR assessment were 5% and 2%, respectively.

### 2.8 Oxidative stress assessment

MDA (nmol/L) was evaluated spectrophotometrically using a commercial kit (Oxford Biomedical Research, Rochester Hills, Michigan, USA) with a colorimetric assay for lipid peroxidation (measurement range: 1–20 nmol/L). For the determination of PCs (nmol/mL), we based this on the spectrophotometric assessment of 2,4–dinitrophenylhydrazine derivatives of PCs according to a previously published methodology.^6^

### 2.9 Statistical analysis and power calculation

The primary endpoint of the present study was the determination of the changes of PBR 4–25 μm after 4 months. The sample size calculation was based on a pilot study with 10 patients in each intervention arm (GDS and placebo). The percentage change of PBR 4–25 μm (ΔPBR%) at 4 months was evaluated for the purpose of the power analysis (GDS: 6.5% and placebo: 1.6%, respectively). To detect a relevant reduction in PBR 4–25 μm with an *α* of .05, 95% power and standard deviation = 4.7, we need at least *n* = 25 subjects per group to carry out this study.

Data were expressed as mean with standard deviation in case of normal distribution or as median with interquartile range in case of non‐normally distributed variables. The Shapiro–Wilk test was implemented in each group separately (GDS and placebo group, pre‐ and post‐treatment) to determine the normality of distribution. Variables with non‐normal distribution were transformed into ranks. Binary variables were presented as numbers with corresponding percentages and were analysed using chi‐square test or Fisher's exact test. The Student's *t* test or Mann–Whitney *U* tests were used to compare continuous variables, as appropriate. We conducted parametric or non‐parametric correlation coefficients tests to detect correlations between the continuous variables. All the analyses were per protocol. Analysis of variance for repeated measurements was carried out for (a) comparisons of the examined markers at baseline and at 4 months of treatment, which was deemed as a within‐subject factor, and (b) for the effects of the type of intervention (GDS or placebo), as a between‐subject factor. The *F* and *p*‐values for the changes in the examined markers at follow‐up assessment were reported. Moreover, the corresponding *F* and *p* values for the interaction between time and intervention group were evaluated. Data analysis was performed using the Statistical Package for Social Sciences (IBM SPSS Statistics for Windows, Version 28.0. Armonk, New York, USA: IBM Corp. Released 2021) and GraphPad Prism version 10.3.0 for Windows (GraphPad Software, Boston, Massachusetts, USA). Statistical significance was set at a two‐tailed *p*‐value <.05.

## 3 RESULTS

### 3.1 Baseline characteristics

Table 1 presents the baseline characteristics of the study population. The mean age of the patients was 56 years, and 58% of them were females. Additionally, 44% of the patients suffered from hypertension, 42% had dyslipidemia, while 39% were current smokers. Patients between the two intervention arms displayed similar demographic, clinical, biochemical, and haematological characteristics at inclusion (*p* > .05; Tables 1 and 2). After 4 months, all patients had increased lymphocytes (*p* < .001) and reduced neutrophils (*p* = .011), monocytes (*p* = .025), neutrophil‐to‐lymphocyte ratio (*p* < .001), monocyte‐to‐lymphocyte ratio (*p* = .003) and platelet‐to‐lymphocyte ratio (*p* = .007). There were no statistically significant differences in the changes of the abovementioned haematological parameters between the two study groups at 4 months of treatment (*p* > .05; Table 2).

**TABLE 1:** Demographic, clinical and biochemical characteristics of the study population.

	All participants (*n* = 57)	GDS (*n* = 29)	Placebo (*n* = 28)	*p*‐Value
Age, years	56 ± 9	55 ± 9	56 ± 8	.664
Female sex, *n* (%)	33 (58)	17 (59)	16 (57)	.774
BMI, kg/m^2^	26.5 ± 4	26.6 ± 4	26.3 ± 3	.790
HbA1c, %	6.6 ± 1	6.7 ± 1	6.4 ± .9	.322
Total cholesterol, mg/dL	195.6 ± 39.3	194.2 ± 37.1	196.8 ± 41.4	.883
LDL‐C, mg/dL	129.2 ± 32.4	128.2 ± 28.4	130.1 ± 36.3	.854
HDL‐C, mg/dL	47.5 ± 15.4	48.6 ± 14.6	46.2 ± 16.1	.678
Triglycerides, mg/dL	116.4 ± 18.1	114.5 ± 16.2	118.2 ± 19.8	.276
SBP, mmHg	136.4 ± 18.4	135.5 ± 17.6	137.2 ± 19.3	.571
DBP, mmHg	79.8 ± 11.1	79.4 ± 11.4	80.3 ± 10.7	.698
Risk factors, *n* (%)				
Hypertension	25 (44)	12 (41)	13 (46)	.774
Dyslipidemia	24 (42)	11 (38)	13 (46)	.367
Diabetes mellitus	13 (23)	7 (24)	6 (21)	.733
Current smoking	22 (39)	12 (41)	10 (36)	.481
Family history of CAD	7 (12)	3 (10)	4 (14)	.637
Medications, *n* (%)				
ACEi/ARB	17 (30)	8 (28)	9 (32)	.758
CCB	13 (23)	6 (21)	7 (25)	.733
β‐Blockers	7 (12)	3 (10)	4 (14)	.637
Diuretics	13 (23)	7 (24)	6 (21)	.733
Statins	24 (42)	11 (38)	13 (46)	.367
Fibrates	1 (2)	0 (0)	1 (4)	.312
Insulin	1 (2)	1 (3)	0 (0)	.312
Antidiabetics	13 (23)	7 (24)	6 (21)	.733

*Note*: Data are presented as mean ± standard deviation or number (percentage). Scale variables were compared using the Student's *t* test. Binary variables were compared with the chi‐square test.

Abbreviations: ACEi, angiotensin‐converting enzyme inhibitors; ARB, angiotensin receptor blockers; BMI, body mass index; CAD, coronary artery disease; CCB, calcium channel blockers; DBP, diastolic blood pressure; GDS, glycocalyx dietary supplement; HbA1c, glycosylated haemoglobin; HDL‐C, high‐density lipoprotein cholesterol; LDL‐C, low‐density lipoprotein cholesterol; SBP, systolic blood pressure.

**TABLE 2:** Changes in biochemical and haematological parameters at 4 months.

	All participants (*n* = 57)	GDS (*n* = 29)	Placebo (*n* = 28)
hs‐CRP (mg/L)			
Baseline	35.5 (16.8–50.5)	35.8 (15.7–59.2)	30.8 (15.1–61.4)
4 months	4.6 (3–5.9)***	4.7 (3.1–6.2)**	4.3 (3–6.2)**
Δ%	−88.2	−88.3	−88.1
eGFR (mL/min/1.73 m^2^)			
Baseline	96 (86–102)	95 (83–100)	96 (88–106)
4 months	96 (86–105)	96 (84–103)	96 (89–107)
Δ%	.7	1.1	.4
Platelets (×10^3^/μL)			
Baseline	259 (205–313)	253 (201–338)	260 (201–299)
4 months	267 (210–301)	267 (207–306)	264 (219–308)
Δ%	5.4	5.1	5.9
White blood cells (×10^3^/μL)			
Baseline	7.66 (6.15–10.56)	7.67 (6.85–9.92)	7.48 (5.77–11.3)
4 months	7.01 (6.26–8.64)	7.3 (6.46–8.94)	6.84 (5.96–7.95)
Δ%	−12.8	−11.8	−13.6
Neutrophils (×10^3^/μL)			
Baseline	6.12 (4.63–8.55)	6.41 (5.32–8.53)	5.53 (4.34–8.84)
4 months	4.4 (3.92–5.72)*	4.72 (4.08–6.3)*	4.08 (3.53–5.24)*
Δ%	−25.5	−24.8	−26.3
Lymphocytes (×10^3^/μL)			
Baseline	1.13 (.77–1.64)	1.05 (.71–1.77)	1.23 (.81–1.69)
4 months	1.85 (1.23–2.44)***	1.72 (1.1–2.55)**	1.88 (1.23–2.25)**
Δ%	52.8	52.9	51.6
Monocytes (×10^3^/μL)			
Baseline	.58 (.47–.73)	.59 (.52–.76)	.57 (.45–.74)
4 months	.49 (.43–.55)*	.48 (.43–.64)*	.5 (.42–.53)*
Δ%	−17.4	−20	−15.5
Neutrophil‐to‐lymphocyte ratio			
Baseline	5.66 (3.95–8.45)	5.91 (4.12–9.12)	4.8 (3.6–7.62)
4 months	2.21 (1.88–3.48)***	2.26 (2.13–4.68)**	2.19 (1.79–3.38)**
Δ%	−51.9	−51.8	−52.1
Monocyte‐to‐lymphocyte ratio			
Baseline	.5 (.39–.79)	.53 (.29–1.07)	.49 (.42–.62)
4 months	.27 (.19–.45)**	.28 (.17–.49)*	.27 (.22–.37)*
Δ%	−47.3	−49.9	−44.5
Platelet‐to‐lymphocyte ratio			
Baseline	227 (142–328)	227 (124–388)	221 (142–326)
4 months	152 (103–215)**	150 (94–252)*	152 (113–179)*
Δ%	−37.3	−40.6	−33.1

*Note*: Data are presented as median (interquartile range). Δ%, percent changes from baseline.

Abbreviations: eGFR, estimated glomerular filtration rate; GDS, glycocalyx dietary supplement; hs‐CRP, high‐sensitivity C‐reactive protein.

*p < .05, **p < .01, ***p < .001 for comparisons of 4 months versus baseline using ANOVA.

At baseline visit (14 days after a confirmed COVID‐19 infection) and in the whole study population, 27 patients (47.3%) presented with COVID‐19 symptoms. In particular, fatigue was present in 23 patients (40.3%), cough in 20 patients (35%), dyspnoea in 11 (19.2%) and chest pain in 6 patients (10.5%). There was no significant difference between the two study arms (GDS and placebo) regarding the percentage of present symptoms (data not shown). At 4 months after COVID‐19 infection and in the placebo group, 6 patients (21.4%) presented with post‐COVID symptoms. Among the symptoms, fatigue was present in 3 patients (10.7%), cough in 2 (7.1%), dyspnoea in 2 (7.1%) and chest pain in 1 patient (3.5%). In the GDS group, none of the patients reported post‐infection symptoms.

### 3.2 Effect of GDS on endothelial glycocalyx integrity

**FIGURE 2:**
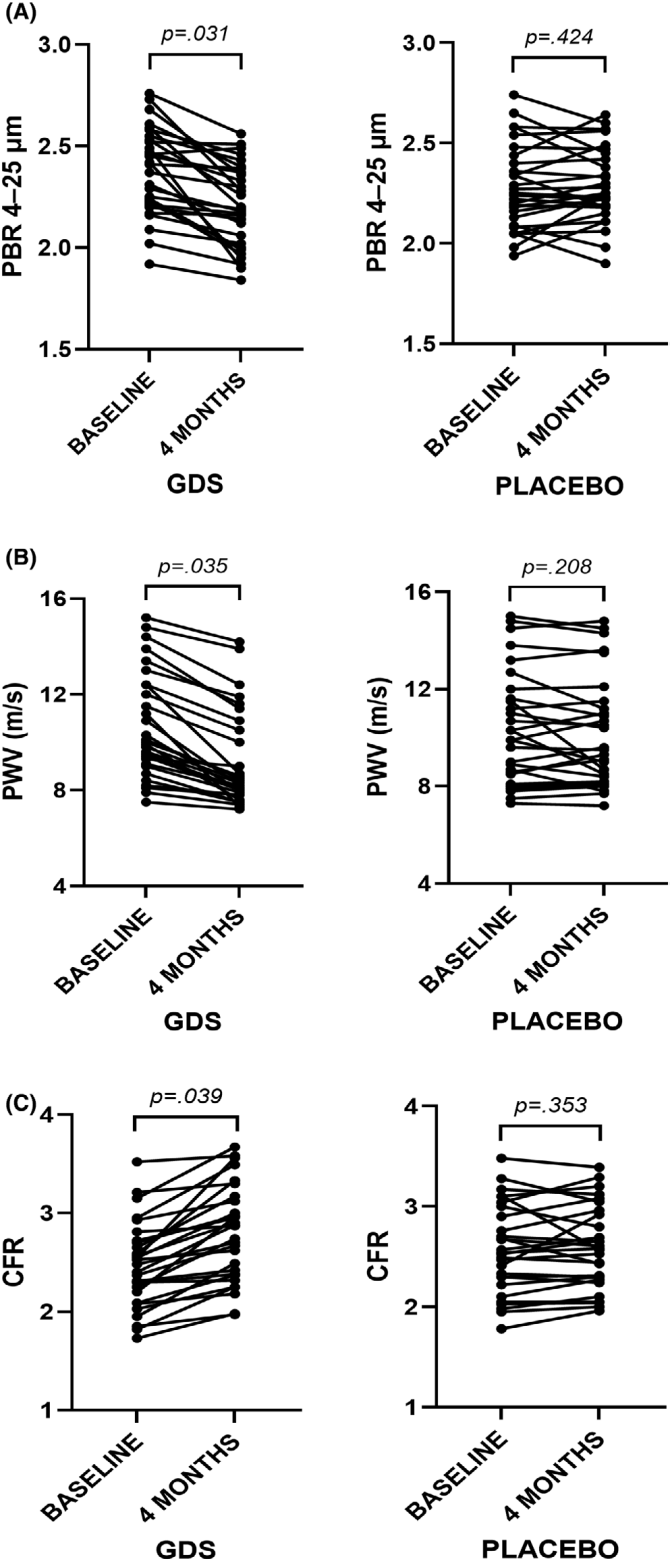
Changes in (A) perfused boundary region (PBR) of the sublingual microvessels with a diameter range of 4–25 μm, (B) pulse wave velocity (PWV) and (C) coronary flow reserve (CFR) after 4 months treatment with glycocalyx dietary supplement (GDS) (*n* = 29) or placebo (*n* = 28).

There were no statistically significant differences in PBR 4–25 μm and total microvascular density between the two groups at baseline (*p* > .05; Table 3). At the 4‐month follow‐up, PBR 4–25 μm reduced significantly from 2.34 ± .22 μm to 2.24 ± .21 μm (*F* = 4.128, *p* = .033), while total microvascular density increased from 182.3 ± 43.1 mm/mm^2^ to 207.9 ± 50 mm/mm^2^ (*F* = 3.458, *p* = .040). Of note, a significant interaction of follow‐up time with the intervention type was observed regarding PBR 4–25 μm (*F* = 5.948, *p* = .016) and total microvascular density (*F* = 3.823, *p* = .039). More specifically, patients who received GDS showed a significant decrease in PBR 4–25 μm (−6.8% vs. −1.3%, *p* = .032, Figure 2A) and a notable increase in total microvascular density (21.7% vs. 6.7%, *p* = .024) compared to the placebo group. A similar trend was observed with regard to PBR 10–19 μm and PBR 20–25 μm (Table 3).

**TABLE 3:** Changes in glycocalyx thickness, arterial stiffness, and oxidative stress markers at 4 months.

	All participants (*n* = 57)	GDS (*n* = 29)	Placebo (*n* = 28)
PBR 4–25 μm			
Baseline	2.34 ± .22	2.37 ± .23	2.30 ± .20
4 months	2.24 ± .21^†^	2.21 ± .20*^,†^	2.27 ± .21
Δ%	−4.3	−6.8	−1.3
PBR 4–9 μm			
Baseline	1.20 ± .09	1.21 ± .07	1.19 ± .10
4 months	1.18 ± .11	1.18 ± .12	1.18 ± .09
Δ%	−1.7	−2.5	−.8
PBR 10–19 μm			
Baseline	2.62 ± .24	2.65 ± .21	2.59 ± .26
4 months	2.54 ± .25^†^	2.52 ± .24*^,†^	2.56 ± .25
Δ%	−3.1	−4.9	−1.2
PBR 20–25 μm			
Baseline	3.01 ± .46	3.04 ± .48	2.96 ± .43
4 months	2.83 ± .43^†††^	2.75 ± .40***^,†††^	2.91 ± .45
Δ%	−5.9	−9.5	−1.7
Total microvascular density (mm/mm^2^)			
Baseline	182.3 ± 43.1	177.8 ± 42.1	186.8 ± 44
4 months	207.9 ± 50^†^	216.4 ± 52.3*^,†^	199.4 ± 47.6
Δ%	14	21.7	6.7
PWV (m/s)			
Baseline	10.5 ± 2.3	10.6 ± 2.2	10.4 ± 2.4
4 months	9.7 ± 2.2^†^	9.2 ± 2.1*^,†^	10.1 ± 2.3
Δ%	−7.6	−13.2	−3
AIx (%)			
Baseline	16.3 ± 5.2	16.5 ± 5.4	16.1 ± 4.9
4 months	13.8 ± 3.9^††^	11.8 ± 3.1*^,††^	15.7 ± 4.4
Δ%	−15.3	−28.5	−2.5
CFR			
Baseline	2.50 ± .45	2.47 ± .45	2.53 ± .44
4 months	2.68 ± .44^†^	2.79 ± .49*^,†^	2.57 ± .39
Δ%	7.2	12.9	1.6
MDA (nmol/L)			
Baseline	1.43 ± .54	1.46 ± .59	1.39 ± .48
4 months	1.22 ± .38^†^	1.08 ± .33*^,†^	1.35 ± .42
Δ%	−14.7	−26	−2.9
PCs (nmol/mL)			
Baseline	13.9 ± 5.43	13.56 ± 5.24	14.13 ± 5.62
4 months	11.66 ± 4.92^††^	9.32 ± 3.66	13.99 ± 6.17
Δ%	−16.2	−31.3**^,††^	−1

*Note*: Data are presented as mean values ± standard deviation. Δ%, percent changes from baseline.

Abbreviations: AIx, augmentation index; CFR, coronary flow reserve; GDS, glycocalyx dietary supplement; MDA, malondialdehyde; PBR, perfused boundary region; PCs, protein carbonyls; PWV, pulse wave velocity.

*p < .05, **p < .01, ***p < .001 for time × treatment interaction obtained by repeated measures ANOVA. ^†^ *p* < .05, ^††^ *p* < .01, ^†††^ *p* < .001 for comparisons of 4 months versus baseline using ANOVA.

### 3.3 Effect of GDS on arterial stiffness markers

The two groups had similar PWV values at baseline (*p* > .05). In all participants, PWV decreased significantly from 10.5 ± 2.3 m/s to 9.7 ± 2.2 m/s (*F* = 3.775, *p* = .042). Furthermore, we noted an important interaction between the change in PWV at follow up and the intervention arm (*F* = 4.188, *p* = .041). At 4 months, patients under GDS achieved a greater reduction in PWV than those under placebo (−13.2% vs. −3%, *p* = .035, Table 3, Figure 2B).

At baseline, AIx values did not differ significantly between the two study groups (*p* > .05). In the total sample, AIx reduced from 16.3 ± 5.2% to 13.8 ± 3.9% (*F* = 6.127, *p* = .008). In addition, a significant interaction between the change in AIx at follow‐up and the intervention arm was observed (*F* = 4.985, *p* = .025). At 4 months, participants under GDS achieved a greater reduction in AIx than those under placebo (−28.5% vs. −2.5%, *p* = .005; Table 3).

### 3.4 Effect of GDS on coronary flow reserve

There were no statistically significant differences regarding CFR between the two groups at baseline (*p* > .05). After 4 months, CFR increased significantly from 2.50 ± .45 to 2.68 ± .44 (*F* = 3.942, *p* = .040). Moreover, a significant interaction of follow‐up time with the intervention type was observed regarding CFR (*F* = 3.749, *p* = .045). More specifically, individuals who received GDS displayed a significant increase in CFR compared to the placebo group (12.9% vs. 1.6%, *p* = .022, Table 3, Figure 2C).

### 3.5 Effect of GDS on oxidative stress markers

The two groups had similar MDA and PCs concentrations at baseline (p > .05). Compared to baseline, all participants had reduced MDA (*F* = 4.875, *p* = .019) and PCs values (*F* = 8.233, *p* = .003) after 4 months of treatment. However, there was a significant interaction between the changes in MDA and PCs at follow‐up and the intervention arm (*F* = 6.697, *p* = .014 and *F* = 7.119, *p* = .004, respectively). At 4 months, patients under GDS achieved a greater reduction in MDA (−26% vs. −2.9%, *p* = .015) and PCs (−31.3% vs. −1%, *p* = .002; Table 3) in comparison with the placebo group.

### 3.6 Associations between endothelial, vascular, and oxidative stress markers

In the whole study population, PBR 4–25 μm and PWV were positively related to high‐sensitivity C‐reactive protein (*r* = .47, *p* = .005 and *r* = .43, *p* = .006, respectively), neutrophil‐to‐lymphocyte ratio (*r* = .33, *p* = .035 and *r* = .30, *p* = .046) and platelet‐to‐lymphocyte ratio (*r* = .31, *p* = .041 and *r* = .37, *p* = .017). Moreover, MDA and PCs levels were associated with PWV (*r* = .32, *p* = .041 and *r* = .35, *p* = .037, respectively), while PCs concentration was correlated with PBR 4–25 μm (*r* = .40, *p* = .028) and CFR (*r* = −.29, *p* = .048).

In the GDS group, the percentage reduction in PBR 4–25 μm was correlated with the respective decrease in PWV (*r* = .31, *p* = .047), MDA (*r* = .39, *p* = .027), and PCs (*r* = .42, *p* = .023) and with the increase in CFR (*r* = −.59, *p* = .008) after the 4‐month intervention.

## 4 DISCUSSION

In this study, we demonstrated that convalescent patients after mild‐to‐moderate COVID‐19 infection managed in an outpatient setting, who were administered GDS for 4 months, showed significant improvement in endothelial glycocalyx integrity, as assessed by PBR and total microvascular density, arterial stiffness, as evaluated by PWV and AIx, and coronary microcirculatory function, as assessed by CFR, in conjunction with a remarkable reduction of oxidative stress markers compared to those who received placebo. Interestingly, in the GDS group, the improvement in PBR was associated with the respective amelioration in PWV, CFR as well as with the decrease in oxidative stress. Thus, short‐term administration of GDS seems to exert beneficial effects on both endothelial and vascular function after COVID‐19 infection. Notably, in contrast to the placebo group, none of the patients who received GDS reported post‐COVID symptoms.

We have previously demonstrated that COVID‐19 patients displayed impaired endothelial glycocalyx, as assessed by greater PBR values, compared to healthy subjects that remained 4 months after initial infection. Increased oxidative burden appeared to be the major contributing factor, as it was 10‐fold higher in comparison with the control group.^3^ Consistent with the aforementioned findings, the current study showed that increased oxidative burden was related to elevated baseline values of PBR and also the percentage decrease in PBR was in direct proportion to the fall in oxidative stress markers after 4‐month treatment with GDS. In addition to oxidative stress, SARS‐CoV‐2‐induced endothelial dysfunction is linked to the inflammatory response. Pro‐inflammatory cytokines, such as tumour necrosis factor‐α, interleukin‐1β and interleukin‐6, lead to changes in vascular permeability resulting in interstitial fluid shift and edema development, as well as in a hypercoagulable endothelial surface.^19^ Furthermore, activated glycocalyx shedding markers, including angiopoietin‐2, heparanase, hyaluronidase, matrix metalloproteinases, and the viral proteins may contribute to the degradation of the endothelial glycocalyx layer in SARS‐CoV‐2 infected patients.^20^, ^21^

**FIGURE 3:**
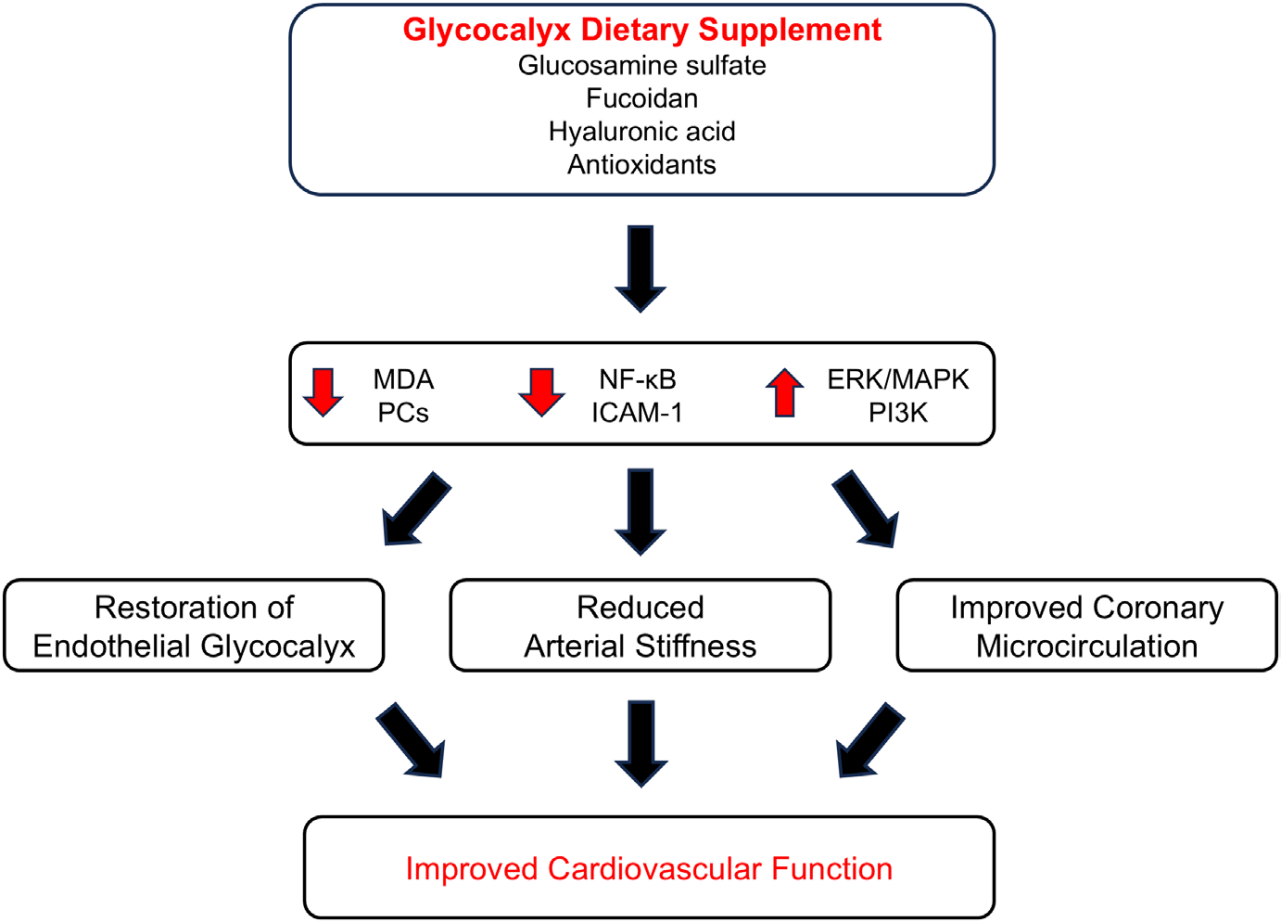
Pathophysiologic mechanisms of glycocalyx dietary supplement (GDS) potential effects on cardiovascular function. GDS appears to reduce oxidative stress markers, including malondialdehyde (MDA) and protein carbonyls (PCs), and decrease endothelial activation through inactivation of the nuclear factor kappa B (NF‐κB) pathway and downstream intercellular adhesion molecule‐1 (ICAM‐1) expression. Moreover, GDS administration improves endothelial glycocalyx via the extracellular signal‐regulated kinase (ERK)/mitogen‐activated protein kinase (MAPK) and phosphatidylinositol 3‐kinase (PI3K) signalling pathways. These mechanisms lead to improved cardiovascular function through the restoration of endothelial glycocalyx, reduction in arterial stiffness, and amelioration in coronary microcirculatory function.

In this study, we showed that 4‐month administration of GDS resulted in essential improvement in endothelial glycocalyx integrity, as evaluated by sublingual PBR measurement and total microvascular density, in subjects after COVID‐19. This finding is in line with a recently published study where treatment with GDS for the same time frame improved glycocalyx integrity in patients with psoriasis.^13^ Glycocalyx dietary supplement provides major substrates for glycocalyx synthesis, including hyaluronic acid, antioxidants, such as superoxide dismutase and polyphenols that protect glycocalyx layer against damage from reactive oxygen species, as well as fucoidan, which is a glycosaminoglycans mimetic.^9^ It has been shown that fucoidan restores glycocalyx components leading to decrease in endothelial activation through inactivation of the nuclear factor kappa B pathway and downstream intercellular adhesion molecule‐1 expression. Thus, fucoidan contributes to the recovery of endothelial barrier function and induces beneficial antithrombotic effects suggesting that glycocalyx preservation may be a potential therapeutic target for controlling acute COVID‐19 injury in hospitalized patients.^20^ In a recent study, endothelial glycocalyx integrity was assessed on living endothelial cells from haemodialysis patients using atomic force microscopy and in mice using intravital microscopy of cremasteric vessels. This study revealed that GDS administration improved endothelial glycocalyx thickness both in vitro and in vivo, mainly through the extracellular signal‐regulated kinase/mitogen‐activated protein kinase and phosphatidylinositol 3‐kinase signalling pathways.^9^ In contrast, Smith et al. found that GDS can lead to improvement in endothelial glycocalyx length and vascular function in diabetic mice, but GDS was not effective after 3‐month administration in individuals with type 2 diabetes mellitus.^22^ However, in another randomized, placebo‐controlled study of subjects with type 2 diabetes mellitus, 3‐month treatment with GDS significantly improved microvascular endothelial health by reducing PBR and the inflammatory cytokine monocyte chemoattractant‐1.^11^ On the other hand, a pilot study suggested that 3‐month GDS administration in older adults did not present measurable effect on overall endothelial glycocalyx thickness. Intriguingly, in the above study, GDS increased capillary glycocalyx thickness in subjects not taking antihypertensive treatment.^12^ Taking into account the aforementioned finding, our study is the first to address the favourable effects of GDS in subjects after mild‐to‐moderate COVID‐19 infection. This supplement seems to counterbalance the detrimental impact of SARS‐CoV‐2 on endothelial function and restore glycocalyx integrity by providing vital components of the endothelial glycocalyx layers (Figure 3).

There is a robust association of COVID‐19 with elevated PWV reflecting a substantial rise in arterial stiffness.^23^ As demonstrated before,^3^ even 4 months after infection, convalescent patients displayed higher PWV values compared to non‐infected control participants. In the present study, we found a significant reduction in arterial stiffness, as assessed by PWV and AIx, in subjects who received GDS compared to those under placebo, when treated 4 months after COVID‐19 infection. This finding may be explained by the favourable effect of GDS on endothelial function. An experimental study showed that 10‐week treatment with GDS in mice resulted in amelioration in PBR and endothelium‐dependent dilation in parallel with the decrease in PWV. These data suggest that the restoration of glycocalyx barrier function mediates more effective shear stress transduction to endothelial cells, stimulating the production of vasodilator nitric oxide, which in turn improves arterial wall function.^10^ Therefore, GDS appears to improve arterial stiffness mainly through improving endothelial function. Indeed, in our study and in the GDS group, a significant correlation between the changes in PBR and PWV was observed. Consequently, endothelial glycocalyx may be a potential therapeutic target to improve vascular dysfunction.

Interestingly enough, in the present study, the pattern of PWV presented a spontaneous mild decrease of 3% at 4 months in the placebo group, showing an opposite trend compared to that Zanoli et al. demonstrated in hospitalized patients with COVID‐19 infection, where PWV tended to decline much more slowly. In fact, both aortic and brachial PWV initially increased at 3–6 months and then partially reverted to baseline values at 48 weeks.^24^ This discrepancy may be partially explained by the fact that our study included patients with mild‐to‐moderate COVID‐19 infection who did not fulfil criteria for hospitalization and, consequently, they displayed a low systemic inflammatory response, as estimated by inflammatory markers such as high‐sensitivity C‐reactive protein. Furthermore, in the placebo group of our study, only 21.4% of patients experienced post‐COVID symptoms at 4 months after infection, whereas Zanoli et al. reported that 69% of patients had symptoms 3–6 months post‐infection, suggesting a persistent low‐grade inflammatory process after the acute phase of COVID‐19 infection causing prolonged vascular dysfunction. In addition, potential residual confounding factors as well as effects of deconditioning related to hospitalization could be involved.^24^

In hospitalized patients, high‐sensitivity C‐reactive protein and neutrophil‐to‐lymphocyte ratio are independent prognostic markers in patients with COVID‐19 infection,^25^, ^26^ while high‐sensitivity C‐reactive protein, estimated glomerular filtration rate and total cholesterol are main determinants of aortic PWV.^24^ Indeed, in the present study, we found that PWV as well as PBR were associated with high‐sensitivity C‐reactive protein and neutrophil‐to‐lymphocyte ratio. Nevertheless, there were no significant differences in the improvement of the abovementioned inflammatory markers between patients who received GDS and those under placebo after 4 months of treatment. This finding suggests that the beneficial effects of GDS on endothelial and vascular function may be mediated by its direct action on endothelial glycocalyx and/or by reducing oxidative stress and not by suppressing the inflammatory process. Actually, in our previous study^3^ oxidative stress appeared the main factor contributing to vascular dysfunction in COVID‐19 patients as it was 10‐fold higher compared to matched hypertensives and normal controls.

The presence of coronary microcirculatory dysfunction is common in patients with COVID‐19 and is associated with the severity of the infection.^3^, ^27^, ^28^ The underlying pathophysiological mechanisms include vascular remodelling, luminal stenosis, increased oxidative stress, impaired endothelial and smooth muscle cell function, and increased sympathetic activity resulting in vasoconstriction and decreased coronary blood flow.^29^, ^30^ Endothelial dysfunction is considered one of the most detrimental pathways of SARS‐CoV‐2‐induced inflammation.^31^ In the present study, we showed a profound increase in CFR in subjects treated with GDS, compared to the placebo group, presumably through the beneficial effect of this supplement on endothelial function and oxidative stress. Of note, in the GDS group, a strong association between the changes in endothelial glycocalyx integrity, oxidative stress markers, and CFR was also documented. Thus, our findings suggest that GDS might contribute to the protection of the coronary microvascular bed in the context of COVID‐19 infection.

Long‐term cardiovascular sequelae after COVID‐19 infection may originate from exacerbation of preexisting established heart disease or as a result of damage that occurred during the acute phase of infection leading to endothelial dysfunction, prothrombotic state, stroke, acute myocardial infarction, myocardial fibrosis, arrhythmias, microvascular disease, ventricular dysfunction, and heart failure.^32^, ^33^ Notably, the risk of cardiovascular complications exists not only in severe COVID‐19 infection but also in individuals who had asymptomatic or mild disease. GDS administration early after SARS‐CoV‐2 infection may be a useful therapeutic option to reduce the risk of long‐term COVID‐19‐associated adverse cardiovascular events by improving the microvascular endothelial function.

### 4.1 Limitations

The limitations of the current study were the relatively small number of participants and the short follow‐up period. Consequently, large‐scale clinical trials are needed to further investigate the long‐term effects of this dietary supplement on cardiovascular function and to corroborate our findings. Despite these limitations, the present study is a randomized, double‐blind, placebo‐controlled trial that provides novel evidence regarding the efficacy of GDS in improving endothelial and vascular function after COVID‐19 infection.

## 5 CONCLUSIONS

In conclusion, in our study we demonstrated that 4‐month treatment with GDS may improve endothelial glycocalyx integrity, vascular function, and coronary microcirculation, in concordance with the reduction of oxidative stress in subjects after mild‐to‐moderate COVID‐19 infection managed in an outpatient setting. These findings suggest that GDS administration might be an effective dietary intervention to mitigate or prevent potential subclinical COVID‐19‐related cardiovascular complications.

## AUTHOR CONTRIBUTIONS

G.P., V.L., and I.I. contributed to the conceptualization and design of the study. A.K., P.E.N., C.C., J.K., G.K., E.M., and E.K. performed the study. G.P., A.K., and K.K. collected data. G.P., J.T., and I.I. analysed the data. G.P. and I.I. drafted the manuscript. J.P., H.V., R.L., S.T., and V.L. critically reviewed the manuscript. All authors read and approved the final manuscript.

## FUNDING INFORMATION

This research received no external funding.

## CONFLICT OF INTEREST STATEMENT

The authors declare no conflict of interest.

## Data Availability

The data that support the findings of this article are available from the corresponding author upon reasonable request.
